# Comprehensive multi-omics analysis of tandem duplicator phenotypes in non-small cell lung cancer

**DOI:** 10.3389/fmed.2025.1556840

**Published:** 2025-06-04

**Authors:** Jie Li, Xinyan Pan, Yanting Bi, Jian Huang, Jun Peng, ChunLing Bai, Jianhong Hou, Yang Chen

**Affiliations:** ^1^Academy of Biomedical Engineering, Kunming Medical University, Kunming, China; ^2^Department of Pathology, The First People’s Hospital of Yunnan Province, Kunming, China; ^3^Human Resources Office, Kunming Medical University, Kunming, China

**Keywords:** tandem duplicator phenotype, non-small cell lung cancer, copy number variance, clinical pathology, chemotherapy

## Abstract

**Introduction:**

The Tandem Duplicator Phenotype (TDP) is a prominent genomic metric prevalent in non-small cell lung cancer (NSCLC).

**Methods:**

Multi-omics samples including DNA copy number, transcriptomics, somatic single-nucleotide variations, clinical data, and cell-line drug sensitivity from TCGA and CCLE are delved into TDP characteristics.

**Results:**

Our thorough analysis reveals that patients with smaller span sizes TDP have a more intricate genome, moderate pathology, enhanced therapeutic response, favorable prognosis, a more active immune system, and higher drug sensitivity. In contrast, those with larger span sizes TDP exhibited opposite trends.

**Discussion:**

The integrated analysis underscores that an in-depth molecular assessment of TDP can offer invaluable insights into its role in cancers. Classification strategy of TDP could recognize the chemotherapy sensitivity of different NSCLC patients.

## Introduction

As high-throughput biological techniques advance, whole-genome sequencing (WGS) and single nucleotide polymorphism (SNP) datasets now allow for a more profound exploration of instability patterns in human genome rearrangements and the identification of cancer-associated copy number variations (1–6). These recurrent patterns lead to genomic instability and further promote tumorigenesis (7–9). Menghi et al. (10, 11) identified a specific structural variation pattern known as the “tandem duplicator phenotype (TDP).” TDP is characterized by tandem duplication events or segmental duplications where chromosome segments, overlapping with one or more genes, can duplicate, positioning adjacently in various configurations. These duplications can encompass all or part of a gene’s regulatory elements, suggesting that TDP could serve as instability-based biomarkers in the human genome (12, 13).

TDP, a unique instability configuration, has been identified in various cancers (14, 15). For instance, research shows that TDP, particularly those with head-to-tail somatic segmental tandem duplications (TDs), are predominantly present in non-small lung cell cancer, breast cancer, ovarian cancer, and endometrial cancers (10, 11, 16–19). Zhou et al. (16) also detected internal tandem duplications in NSCLC linked to focal adhesion kinase anomalies. These duplications augmented the autophosphorylation proteins level and heightened sensitivity to FAK inhibitors. However, most previous NSCLC studies introduced genomic fingerprints of NSCLC, active immune landscape appears in NSCLC patients with high mutation burden, these multiple somatic mutation gene are associated with DNA repair (20–24). Several studies have demonstrated that *EGFR* kinase domain duplication typically results from an in-frame tandem duplication of exons 18–25, leading to constitutive activation of *EGFR* signaling via the formation of an intramolecular dimer in non-small cell lung cancer (NSCLC). Similarly, *BRAF* rearrangements, including kinase domain duplications, have been identified at a frequency of approximately 4.3%. These findings emphasize the clinical relevance of *EGFR* and *BRAF* kinase domain duplications in NSCLC and highlight the potential of targeted therapies tailored to these recurrent molecular alterations (25–27), the impact of varying TD lengths on tumor progression is yet to be fully understood. Thus, comprehensive investigations considering TDP effects on CNV, clinical variations, transcripts, mutation characteristics, and drug sensitivity are warranted (28–30).

In this study, we developed TDP models for NSCLC, encompassing both lung adenocarcinoma (LUAD) and lung squamous cell carcinoma (LUSC), by curating existing SNP data from the TCGA and CCLE cohorts (31–33). Our goal is to deeply characterize the molecular attributes of TDP and methodically examine their functions. We also present a comprehensive analysis of clinical variations and drug sensitivity related to TDP. This suggests the prospective utility of TDP groups as predictive genomic biomarkers in clinical scenarios.

## Methods

### Data collection

To comprehensively characterize somatic tandem duplications (TDs) in NSCLC, including LUAD and LUSC, we amassed diverse genomic datasets. These encompass masked copy number variation (CNV) data, transcriptomics, somatic mutations, and drug sensitivity metrics from the Cancer Genome Atlas (TCGA) and the Cancer Cell Line Encyclopedia (CCLE) (31–33). We derived the masked CNV data from the Affymetrix Genome-Wide Human SNP Array 6.0, utilizing it to identify TDs. To further delineate the distinct features of TDP groups, we utilized transcript and somatic mutation datasets from TCGA. Pertinent clinical data, including relapse events and timelines for TCGA patients, were sourced from the UCSC Cancer Genomic Browser.^1^

### TDP classification

Utilizing the masked CNV data, we set criteria to define tandem duplication segments based on three rules. Firstly, the amplification CN segment exceeded 100 bp in length; Secondly, the segment exhibited an increase relative to adjacent segments, with a log2 CN ratio of at least 0.3; Thirdly, differences between consecutive neighboring segments maintained a log2 CN ratio of 0.3 or less. Following these criteria, we determined a TDP score for each tumor sample, derived from the count and chromosomal distribution of somatic tandem duplications (TDs). As per this metric,


$$\mathrm{Raw}\;\mathrm{TDP}\;\text{score}=-\frac{\sum_{i}|{\mathrm{Obs}}_{i}-\exp_{i}|}{\mathrm{TD}},$$

where TD represented the total number of TDs in a sample, ${\mathrm{Obs}}_{i}$ represented the observed number of TDs of chromosome i, $\exp_{i}$ represented the expected number of TDs of chromosome i across all analysis samples, and i represented chromosome 1 to 22 and chromosome X.

Samples with raw TDP scores below the mean value of the first mode in the Gaussian finite distribution, or those with fewer than 20 TDs, were categorized into the non-TDP group. In contrast, samples with TDP scores exceeding the mean value of the second mode in the Gaussian finite distribution were identified as part of the TDP group, in alignment with the approach taken in the previous study by Menghi et al. (10).

In the TDP group, we analyzed the span size of the density distribution for all detected TDs (34, 35). Notably, the distribution peaks in each TDP group predominantly clustered at recurrent and distinct span-size intervals. To discern the pattern of these distribution peaks, we applied the “mclust” R package for Gaussian fitting distribution (25, 36–38). This helped determine the optimal number of mixture components, utilizing default values for the initialization of the iterative process. In each cohort, by marking thresholds at intersections between consecutive Gaussian curves, we distinguished span size ranges across distinct, non-overlapping intervals. Previous study used TDP group 1 (tens of base pairs), TDP group 2 (hundreds of base pairs), TDP group 3 (> 1.7 mb base pairs) to classify TDP class (19). We define the similar criterion in our TDP group in LUAD and LUSC. This process led to the definition of six unique TDP groups based on different intervals. Of these, TDP group 1, TDP group 1/2 mix, and TDP group 1/3 mix were categorized as small span size groups (SSG).

### Clinical information collection and prognostic analysis

We subsequently compared the clinical information across various TDP groups. For the TCGA LUAD cohort, we collected data on clinical stage, distal metastasis stage, and therapy response for analysis. In the TCGA LUSC cohort, we examined the clinical stage, lymph node stage, and therapy response. Additionally, we employed the Kaplan–Meier survival analysis and the log-rank test to compare recurrence-free survival among the different TDP groups.

### Analysis of gene differential expression

We selected two TDP groups, TDP group 2 and TDP group 2/3 mix, which exhibited notably different clinical information and had a larger patient count for the analysis of transcript differences. Within the TCGA LUAD cohort, we examined both TDP group 2 and TDP group 2/3 mix patients. For the TCGA LUSC cohort, we focused on SSG and TDP group 2/3 mix patients. We employed the R package “limma” to determine differentially expressed genes in the FPKM files, and the “quantile” function from “limma” was used for normalization. R package “limma” used empirical bayes to smooth of standard errors, (shrinks standard errors that are much larger or smaller than those from other genes towards the average standard error), and then used t-test to compare the difference between two groups. In the LUAD datasets, differentially expressed genes were identified with criteria of an adjusted *p*-value <0.01 (BH multiple hypotheses testing) and an absolute fold change ≥2. For the LUSC datasets, the criteria were a *p*-value <0.001 (39). To further explore the KEGG pathways within distinct TDP groups, we used the R package “clusterProfiler” to identify relevant KEGG pathways with a *p*-value threshold of <0.05 (40–42).

### Somatic copy-number analysis

We utilized the Genomic Identification of Significant Targets in Cancer (GISTIC 2.0) to pinpoint target genes within focal somatic copy-number variants (SCNVs) and to identify significant amplification regions containing genes across the six distinct TDP groups (43). For this analysis, we adopted the hg19 reference genome. The SNP annotation markers file was sourced from the UCSC Genome Browser.^2^ Within GISTIC 2.0, the default settings for determining gene amplification or deletion were set with a threshold of 0.1. We used a *q*-value cutoff of ≤0.05, and the confidence level for identifying driver aberrations was established at 90%.

### TD-impacted cancer genes analysis

From the genes identified with amplifications by GISTIC 2.0, we retained only those classified as cancer genes for further analysis. These cancer genes were categorized into tumor suppressor genes (TSGs) and oncogenes (OGs) as proposed by Davoli et al. (44). We selected 300 TSGs and 250 OGs for evaluation, consistent with a prior study. TD-impacted cancer genes were recognized if their genomic locations overlapped with one or more TDs and met any of the following criteria, (i) duplication (DUP): the TD spanned the entire gene body, resulting in gene duplication; (ii) double transection (DT): both TD breakpoints were located within the gene body, leading to a disruption of gene integrity or (iii) single transection (ST): only one TD breakpoint was situated within the gene body, causing an effective gene copy number neutral rearrangement. We then assessed the TD-impacted cancer genes within the distinct TDP groups of each cohort.

### Somatic single-nucleotide variation analysis

We utilized the MutSigCV2.0 algorithm to pinpoint genes that were significantly and recurrently mutated in various TDP groups (45), setting a *p*-value threshold of <0.05. The types of somatic mutations in these genes including synonymous, missense, frame insertion/deletion, frame shift, nonsense, splice sites, and other non-synonymous variants-were visualized across distinct TDP groups using the “pheatmap” R package.

### Drug sensitivity analyses

Based on the somatic copy-number profiles sourced from CCLE, we also categorized NSCLC cell lines into six distinct TDP groups (31). Subsequently, we assessed the variations in drug sensitivity across these TDP groups. We compared IC50 values between the groups when subjected to treatment with 24 different anticancer drugs.

## Results

### TDP patient identification and subtypes classification

**Figure 1:**
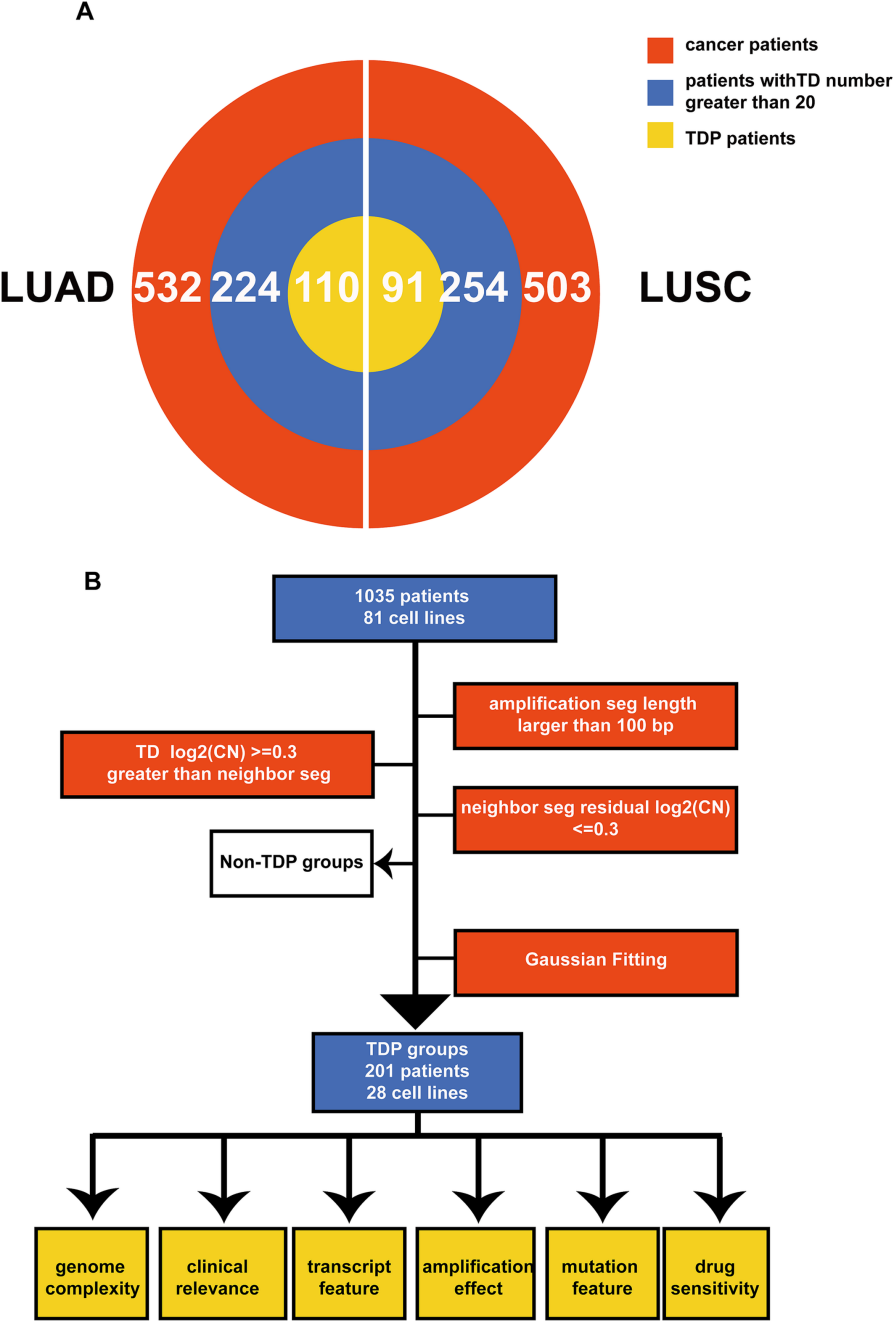
Schematic overview and multi-omics summary of the study. **(A)** The pie chart illustrates the patients chosen for the TDP study from TCGA LUAD and LUSC. The red section represents all patients under analysis, the blue segment denotes patients with more than 20 TDs, and the yellow section signifies those classified as TDP patients. **(B)** This outlines the sequence of operations for classifying TDP patients and the subsequent analyses, which encompass genome complexity, clinical implications, transcriptomic attributes, amplification effects, mutation characteristics, and drug responsiveness.

The tandem duplicator phenotype (TDP) is prevalent in NSCLC, encompassing both LUAD and LUSC. To comprehensively delineate somatic TDP, we sourced and analyzed data from the Affymetrix Genome-Wide Human SNP Array 6.0. This dataset consisted of 1,035 samples and 81 cell lines, which include 532 masked CNV segment data from TCGA LUAD, 503 from TCGA LUSC, and 81 from CCLE NSCLC (Figures 1A,B) (31–33). Through the preprocessing of the SNP array data (Figure 1B), we identified appropriate TDs based on the following criteria. Firstly, we selected amplification segments with lengths exceeding 100 bp. Secondly, the log-transformed CNV ratios of somatic tandem duplications (TDs) surpassed 0.3 when compared to adjacent segments. Thirdly, the difference in log-transformed CNV ratios between two neighboring segments was less than 0.3.

**Figure 2:**
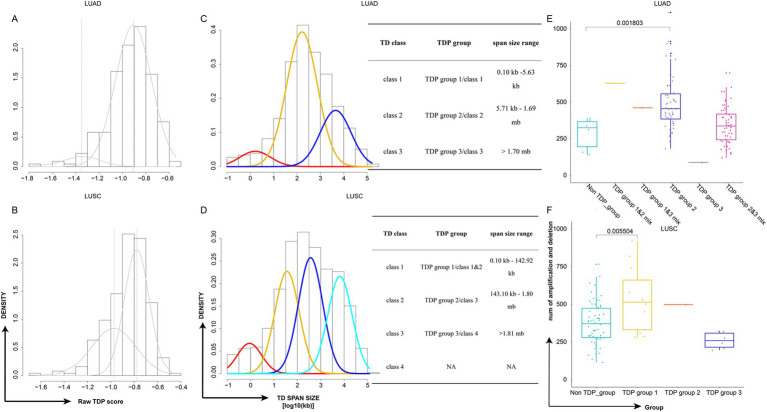
Classification of TDP patient subtypes and their genome complexity. **(A,B)** Density plots depict the raw TDP scores for TCGA LUAD **(A)** and TCGA LUSC **(B)**. **(C,D)** Density plots showcase the TD span size (log10 transformed) for TCGA LUAD **(C)** and TCGA LUSC **(D)**. Accompanying tables detail the classes, groups, and span size ranges. The TD class is determined by the mode in the Gaussian finite distribution. LUAD has three modes, resulting in three TD classes, while LUSC has four modes, leading to four TD classes based on the distribution. **(E,F)** Graphs display the distribution of genome complexity across different TDP groups in TCGA LUAD **(E)** and TCGA LUSC **(F)**, *p*-value was calculated by two-sided Wilcoxon test.

For detection of the number and chromosomal distribution of TDs in cancer patients’ genomes, we used the raw TDP score to characterize this feature (described in methods) (Figures 2A,B). We removed the samples whose TD counts were less than 20 (Figure 1A), and the remaining samples’ raw TDP scores exceeded the mean value of the second mode according to previous study. Subsequently, 110 TDP patients from TCGA LUAD cohort; 91 TDP patients from TCGA LUSC cohort; 28 TDP cell lines from CCLE NSCLC cohort were recognized (Figure 1B).

The “Mclust” function from “Mclust” R package was applied to the TD span size distribution of TDP patients using default parameters. The TD class is defined by the mode in the Gaussian finite distribution. LUAD has three modes, resulting in three TD classes, while LUSC has four modes, leading to four TD classes according to the distribution. We identified three primary TDP groups: LUAD’s first mode and LUSC’s first and second modes as TDP group 1, LUAD’s second mode and LUSC’s third mode as TDP group 2, and LUAD’s third mode and LUSC’s fourth mode as TDP group 3; tumors with bimodal distributions were labeled as TDP group mixes (1/2, 1/3, or 2/3), and notably, TDP group 1, TDP group 1/2 mix, and TDP group 1/3 mix were classified as SSG (Figures 2C,D). The sample counts for TDP groups 1, 2, 3, as well as 1/2, 1/3, and 2/3 mixtures are calculated in TCGA LUAD and LUSC patients (Table 1).

**Table 1:** Number of TDP groups in TCGA LUAD and LUSC.

TDP classification	LUAD	LUSC
TDP group 1	0	11
TDP group 1/2 mix	1	31
TDP group 1/3 mix	1	16
TDP group 2	52	1
TDP group 3	1	6
TDP group 2/3 mix	55	24

Distinct model distribution patterns often correlate with varying genome complexities. Thus, we computed the genome complexities for the three primary TDP groups and non-TDP groups from the aforementioned datasets. It emerged that the non-TDP group exhibited significantly fewer amplification and deletion regions compared to the TDP groups, with TDP group 1 and TDP group 2 exhibiting the most pronounced genome complexity (Figures 2E,F).

### Clinical association and prognosis difference in distinct TDP groups

Genomic instability is a recognized hallmark of cancer based on previous research. However, the relationship between TDP and cancer remains elusive. To bridge this gap, we explored the association between patient clinical data and TDP groups and evaluated the prognosis outcomes across these groups.

**Figure 3:**
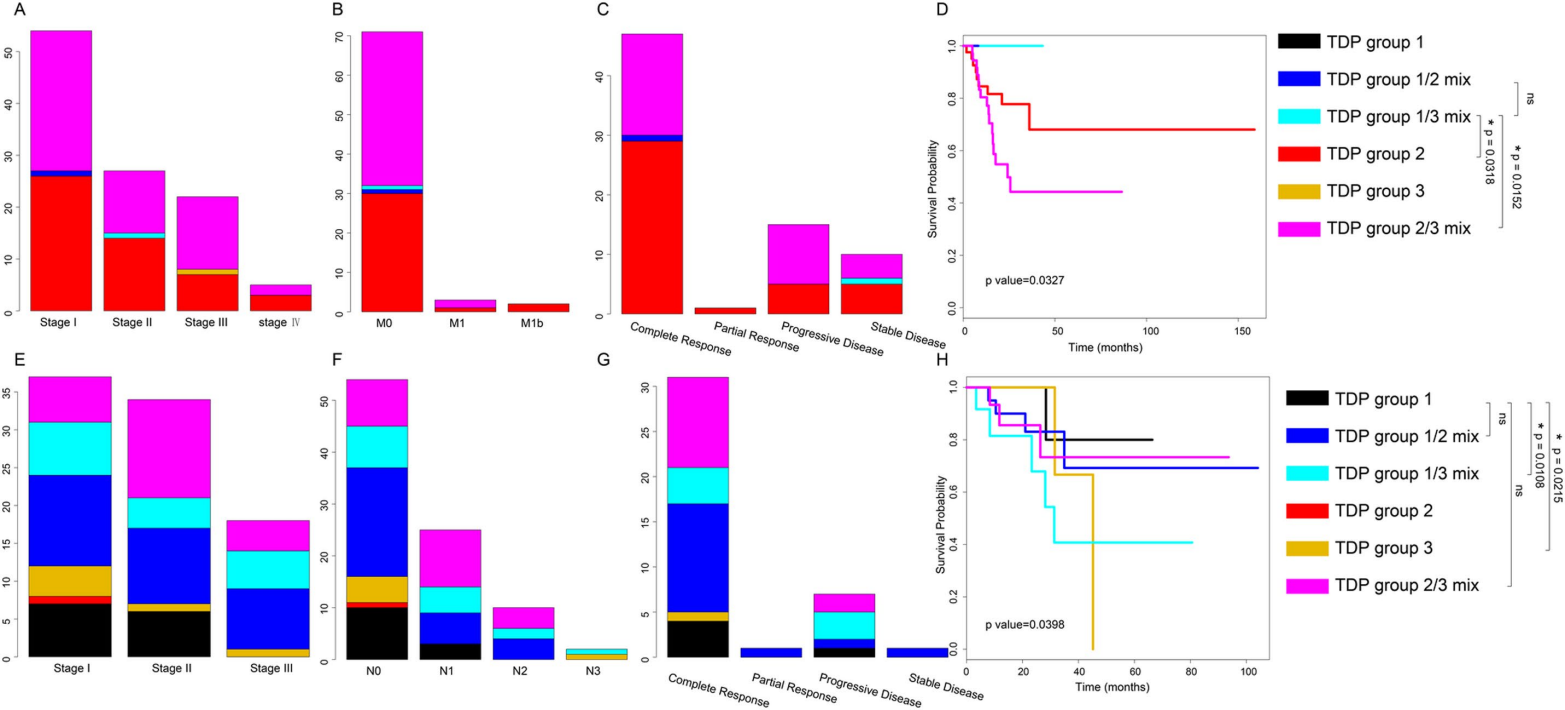
Distribution of clinical data and prognosis across various TDP groups. **(A–C)** Graphs illustrate the distribution of clinical stage **(A)**, distal metastasis **(B)**, and therapy response **(C)** among TDP group patients within the TCGA LUAD dataset. **(D)** Kaplan–Meier survival curves and log-rank tests were executed for the TCGA LUAD dataset. TDP group 1/3 mix patients have better prognosis outcome than TDP group 2 patients (*p*-value = 0.0318), TDP group 1/3 mix patients have better prognosis outcome than TDP group 2/3 patients (*p*-value = 0.0152). **(E–G)** Charts depict the distribution of clinical stage **(E)**, lymph node stage **(F)**, and therapy response **(G)** for patients in different TDP groups within the TCGA LUSC dataset. **(H)** Kaplan–Meier survival curves and log-rank tests were conducted for the TCGA LUSC dataset. TDP group 1 patients have better prognosis outcome than TDP group 1/3 patients (*p*-value = 0.0108), TDP group 1 patients have better prognosis outcome than TDP group 3 patients (*p*-value = 0.0215).

For TCGA LUAD patients, our findings indicated that patients in the TDP group 1/2 mix and TDP group 1/3 mix had a relatively moderate clinical stage (Figure 3A). They displayed no distal metastasis (Figure 3B), and notably, the TDP group 1/2 mix patients showed a complete therapy response (Figure 3C). On the other hand, a section of the patients in TDP group 2 and TDP group 2/3 mix exhibited severe clinical stages, manifested metastasis, and had an unsatisfactory therapy response (Figures 3A–C). In terms of relapse-free survival time, patients from TDP group 1/2 mix and TDP group 1/3 mix significantly had better outcome than those from TDP group 2 and TDP group 2/3 mix, with a log-rank test *p*-value of 0.0327 (Figure 3D).

For TCGA LUSC patients, those in TDP group 1 predominantly exhibited clinical stages of I and II (Figure 3E). The majority showed no lymph node metastasis and had a full therapy response (Figures 3F,G). However, patients from other groups, in contrast, had a relatively severe clinical stage, demonstrated lymph node metastasis, and responded poorly to therapy (Figures 3E–G). Remarkably, TDP group 1 patients exhibited significantly improved outcomes concerning relapse-free survival time, with a log-rank test *p*-value of 0.0398 (Figure 3H).

Drawing conclusions from the clinical associations and prognostic analyses across different TDP groups, it’s evident that patients in TDP group 1 and SSG displayed moderate pathology, superior therapy response, and a more favorable prognosis compared to their counterparts in the TDP group 2/3 mix.

### Smaller span size group suggested active immune system

**Figure 4:**
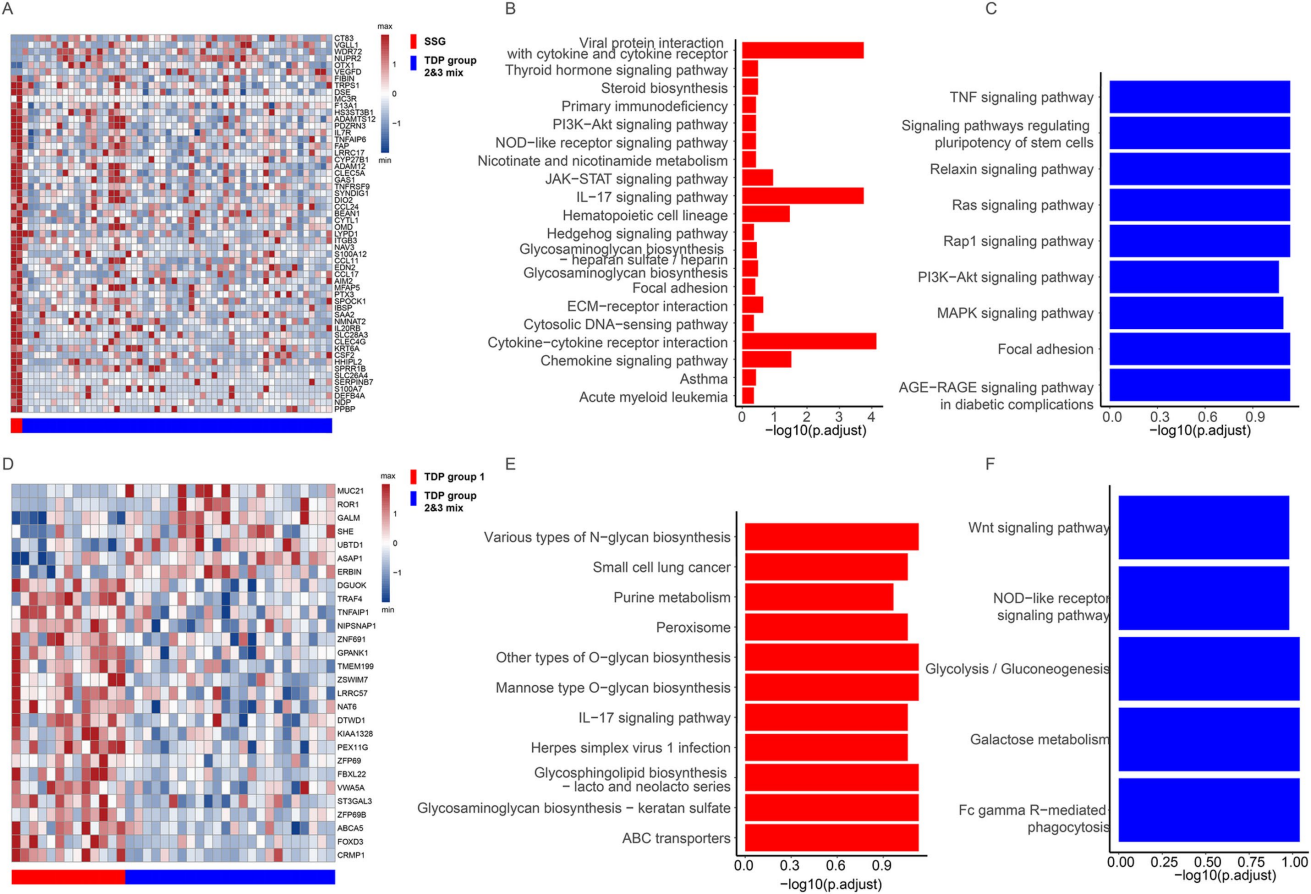
Transcriptional features across different TDP groups. **(A)** Differentially expressed genes are showcased between SSG and TDP group 2/3 mix in TCGA LUAD patients. **(B,C)** Charts depict pathways that are upregulated in SSG **(B)** compared to those in TDP group 2/3 mix **(C)** of TCGA LUAD patients. **(D)** Illustration of the differentially expressed genes between TDP group 1 and TDP group 2/3 mix in TCGA LUSC patients. **(E,F)** Graphs highlight pathways that are upregulated in TDP group 1 **(E)** versus those in TDP group 2/3 mix **(F)** of TCGA LUSC patients.

To delve deeper into why different TDP groups exhibit varied pathology, we compared transcript differences between SSG patients (with moderate pathology) and TDP group 2/3 mix patients (with severe pathology) in LUAD. We identified 50 genes with elevated expression in SSG patients (Figure 4A). Enriched KEGG pathways related to cell proliferation and migration included “PI3K-Akt signaling pathway,” “JAK-STAT signaling pathway,” “Focal adhesion,” and “ECM-receptor interaction.” Notably, immune pathways like “IL-17 signaling pathway,” “Cytokine-cytokine receptor interaction,” and “Chemokine signaling pathway” were also enriched (Figure 4B). The activation of these immune pathways suggests that SSG patients possess an active immune response, potentially contributing to their moderate pathology. Conversely, six genes showed heightened expression in TDP group 2/3 mix patients (Figure 4A), with cell proliferation and migration pathways such as “PI3K-Akt signaling pathway,” “MAPK signaling pathway,” “Ras signaling pathway,” “Rap1 signaling pathway,” and “TNF signaling pathway” being enriched (Figure 4C), possibly driving the severe pathology observed.

In TCGA LUSC patients, we discerned transcript differences between TDP group 1 and TDP group 2/3 mix patients. Twenty-one genes were found to have increased expression in TDP group 1 patients (Figure 4D). The “IL-17 signaling pathway” was similarly enriched as observed in LUAD SSG patients, accompanied by activation of numerous metabolic pathways (Figure 4E). In contrast, seven genes exhibited heightened expression in TDP group 2/3 mix patients (Figure 4D), with the enrichment of pathways like “Wnt signaling pathway,” “Glycolysis/Gluconeogenesis,” and “Galactose metabolism” (Figure 4F).

In summary, the activation of immune pathways in SSG and TDP group 1 patients may underpin their moderate pathology. Conversely, the prominence of cell proliferation and migration pathways in TDP group 2/3 mix patients could be responsible for their severe pathology.

### Cancer gene amplification effect in distinct TDP groups

Following our exploration into TDP groups, we further investigated how TDs influence oncogenes (OGs) and tumor suppressor genes (TSGs). For this purpose, we relied on 300 TSGs and 250 OGs classified by Davoli et al. (44) for an in-depth analysis, TD target genes were pinpointed using GISTIC 2.0 (43). As delineated in the methods, TDs can impact the integrity of a gene’s body in three main ways: (1) duplication (DUP) leading to gene replication, (2) double transection (DT) causing gene disruptions, and (3) single transection (ST) that results in an effective gene copy-number neutral rearrangement.

For the sake of robust results and considering sample size, our focus turned to TDP groups with substantial numbers—specifically, TDP group 2 and TDP group 2/3 mix in LUAD, along with SSG and TDP group 2/3 mix in LUSC—to examine the effects of cancer gene amplification. Amplification regions were identified by GISTIC 2.0, and genes from these regions with a *p*-value below 0.05 were selected for further scrutiny.

**Figure 5:**
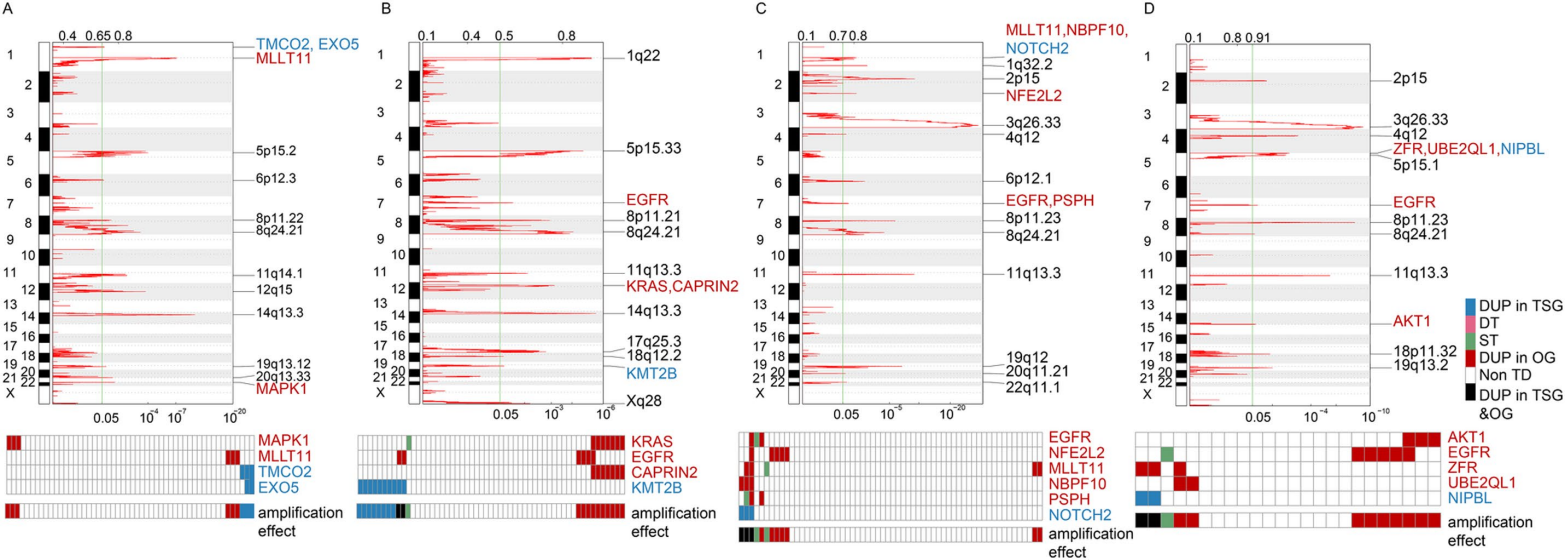
Amplification effects on cancer genes across different TDP groups. **(A,B)** Graphs represent the amplification effects on OGs and TSGs in TDP group 2 **(A)** compared to TDP group 2/3 mix **(B)** for TCGA LUAD patients. **(C,D)** Illustrations depict the amplification effects on OGs and TSGs in SSG **(C)** versus TDP group 2/3 mix **(D)** for TCGA LUSC patients.

Our findings highlighted that in LUAD TDP group 2 patients, OGs like “MAPK1” and “MLLT11” along with TSGs such as “TMCO2” and “EXO5” were subject to the DUP effect. Specifically, the DUP effect for OGs was observed in 6 out of 52 TDP group 2 patients, and for TSGs, this effect was noted in 4 out of the same 52 patients (Figure 5A). For the LUAD TDP group 2/3 mix, OGs like “KRAS,” “EGFR,” and “CAPRIN2” and TSGs including “KMT2B” manifested the DUP effect. Here, the DUP effect was present in 10 of 55 patients for OGs and 8 of 55 for TSGs (Figure 5B). These amplifications, particularly in OGs, could potentially underlie the severe pathology.

For the TCGA LUSC dataset, OGs such as “EGFR,” “NFE2L2,” “MLLT11,” “NBPF10,” and “PSPH” as well as TSGs like “NOTCH2” exhibited the DUP effect in SSG patients, with the OGs’ DUP effect recorded in 7 out of 60 SSG patients (Figure 5C). In the TDP group 2/3 mix patients, OGs including “AKT1,” “EGFR,” “ZFR,” and “UBE2QL1” and TSGs like “NIPBL” demonstrated the DUP effect. Here, the OGs’ DUP effect was evident in 9 out of the 24 TDP group 2/3 mix patients (Figure 5D). This higher proportion of affected patients underscores the link between severe pathology and the TDP group 2/3 mix.

### Insights into mutation feature in distinct TDP groups

**Figure 6:**
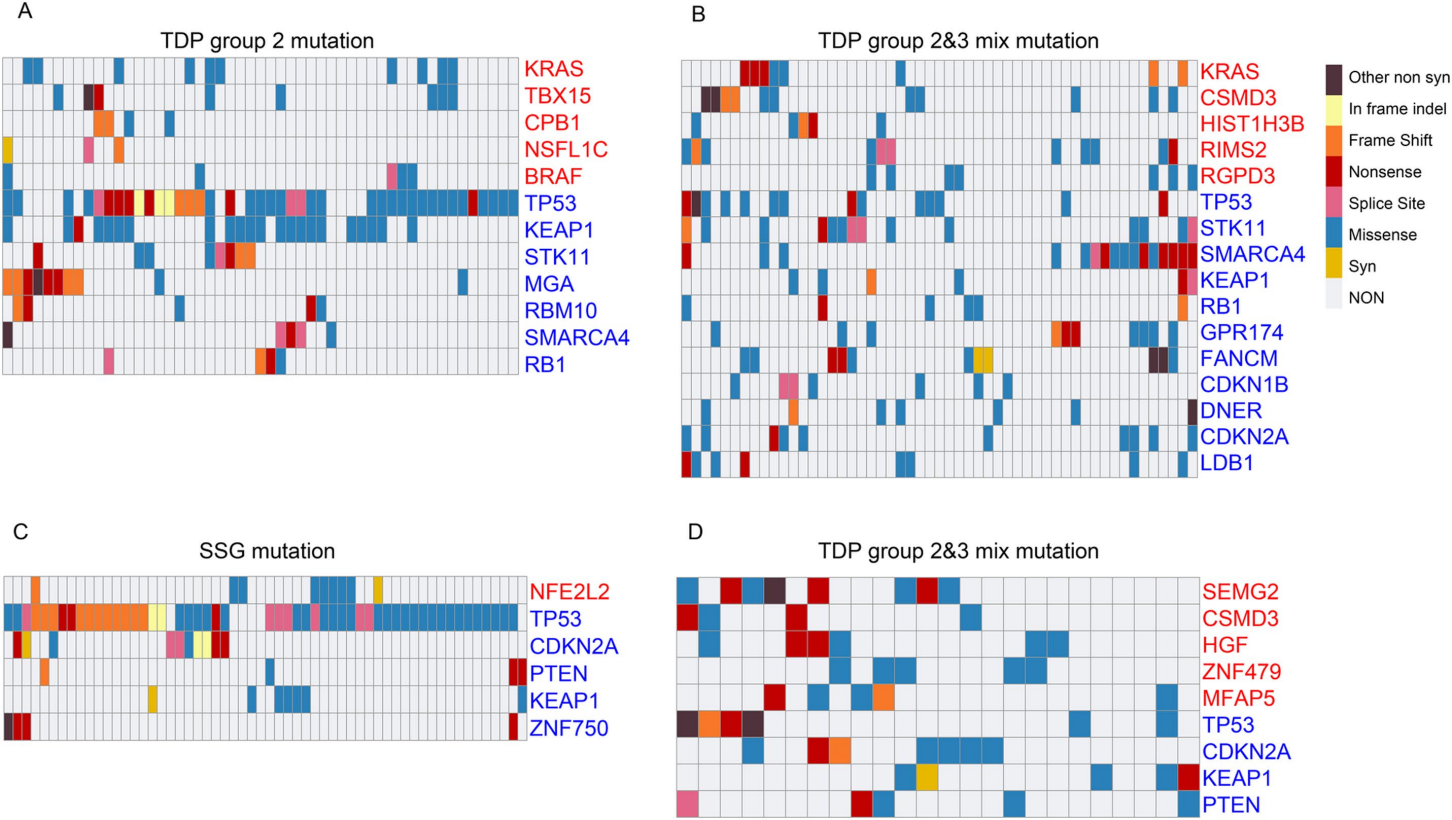
Mutation characteristics of cancer genes across different TDP groups. **(A,B)** The mutation patterns of OGs and TSGs are displayed for TDP group 2 **(A)** in comparison to TDP group 2/3 mix **(B)** in TCGA LUAD patients. **(C,D)** Charts showcase the mutation characteristics of OGs and TSGs for SSG **(C)** versus TDP group 2/3 mix **(D)** in TCGA LUSC patients.

We further examined the mutation characteristics of OGs and TSGs across various TDP groups. Among TCGA LUAD patients, commonly occurring mutations of OG “KRAS” and TSGs such as “TP53,” “KEAP1,” “RB1,” and “SMARCA4” were identified in both TDP group 2 and TDP group 2/3 mix patients. Additionally, each group presented with its unique set of mutated genes (Figures 6A,B). Previous research by Ambrogio et al. (46) highlighted that “KRAS” dimerization enhances the survival of both human and murine KRAS mutant LUAD tumor cells possessing wild-type KRAS, explaining the observed resistance to MEK inhibition. Further, Romero et al. (47) suggested a link between Keap1 loss and KRAS-driven lung cancer.

For TCGA LUSC patients, recurring mutations in TSGs like “TP53,” “CDKN2A,” “PTEN,” and “KEAP1” were observed in both SSG and TDP group 2/3 mix patients. Specifically, mutations in “NFE2L2” and “ZNF750” were detected in SSG patients, while “SEMG2,” “CSMD3,” “HGF,” “ZNF479,” and “MFAP5” mutations were noted in the TDP group 2/3 mix patients (Figures 6C,D). A study by Zhang et al. (48) identified novel genetic disruptions in squamous cell lung carcinoma, pointing out that “CSMD3” is associated with a high frequency of single nucleotide variants.

### Smaller span size TDP groups have greater drug sensitivity

To delve deeper into the varied drug sensitivities across different TDP groups, we sourced pharmacological profiles and segmented copy-number profiles for 24 anticancer drugs, encompassing 504 cell lines from the Cancer Cell Line Encyclopedia^3^ (31).

For the CCLE LUAD cohort, we segmented cell lines into distinct TDP groups using criteria consistent with the TCGA cohort. Upon calculating the TDP score (Supplementary Figures 1A,B) and evaluating the Gaussian fitting distribution (Supplementary Figures 1C,D), we classified cell lines into TDP group 1 and TDP group 1/2 mix. The former comprised six samples, while the latter included twenty-two. Aligning TDP groups with drug therapy concentration data, we noted that following Lapatinib treatment (targeting the “EGFR” gene), the IC50 for TDP group 1/2 mix cell lines was considerably higher than for TDP group 1 cell lines, yielding a *p*-value of 0.0107 (one-sided Wilcoxon test) (Supplementary Figure 1E). A parallel outcome emerged when LUAD cell lines were treated with Panobinostat (targeting the “HDAC” gene), where the *p*-value was 0.0318 (Supplementary Figure 1F).

A comparable analysis was executed for CCLE LUSC cell lines treated with AZD6244 (targeting the “MEK” gene). This resulted in the identification of four TDP group 1 cell lines and seventeen TDP group 1/2 mix cell lines. The IC50 of the TDP group 1/2 mix cell lines was markedly higher than that of the TDP group 1 cell lines, with a *p*-value of 0.0333 (one-sided Wilcoxon test) (Supplementary Figure 1G).

Conclusively, TDP groups with smaller span sizes exhibited lower IC50 values, signifying that their drug sensitivity surpassed that of groups with larger span sizes.

## Discussion

Recurrent genomic rearrangements, such as duplications, translocations, inversions, and deletions, have become a focal point in current research, specifically in the context of TDP. This article explores of TDP, identifying six unique TDP groups based on TD span size, each with distinct characteristics. Notably, TDP group 1 and SSG stand out as exemplars of genomic complexity, while the non-TDP group exhibits the least complexity. Referring to Macintyre’s et al. (49) research, we draw connections between high genomic complexities, particularly CN signatures 1, 7, and 8, and favorable prognoses, with CN signature 7 indicating a tandem duplicator phenotype.

As expected, both TDP group 1 and SSG patients experience significantly improved recurrence-free survival, characterized by a moderate clinical stage, primarily “stage I” and “stage II” absence of distal metastases, moderate lymph node stages, and positive therapy response. In contrast, a substantial portion of TDP group 2/3 mix patients presents severe pathology, evident metastasis, advanced lymph node stages, and less responsive therapy outcomes. We propose that the length of TDP may serve as a potential biomarker for predicting lung cancer recurrence. Considering the clinical relevance of TDP in oncogenes such as EGFR and BRAF, targeted sequencing probes could be developed to detect these alterations more effectively, thereby enabling a more accurate assessment of patient prognosis.

Examining the clinical pathology at the transcriptomic level across different TDP groups reveals that SSG and TDP group 1 patients activate numerous immune-associated pathways, potentially curbing tumor cell growth and spread. Conversely, the TDP group 2/3 mix shows an enrichment of pathways driving cell growth and mobility, likely contributing to their severe pathology.

Analyzing gene amplification in the TDP group 2/3 mix highlights OG duplication, particularly emphasizing the frequent occurrence of EGFR in TCGA LUAD and LUSC cohorts. EGFR amplification, recognized for spurring cell proliferation, may hinder targeted treatments, especially in tumors with EGFR-Kinase domain duplications (26, 50). Notably, TDP group 2/3 mix patients exhibit a worse prognosis, possibly due to the amplification of EGFR wild-type alleles.

On the mutation front, recurrent “KRAS” mutations are identified in both TDP group 2 and TDP group 2/3 mix of TCGA LUAD, suggesting potential resistance and adverse pathology. Similarly, the “CSMD3” mutation, a driver of LUSC, is prevalent in the TDP group 2/3 mix, contributing to severe pathology.

Neo-adjuvant studies underscore the responsiveness of TDP group 1 cell lines to treatments, with lower IC50 values than TDP group 1/2 mix cell lines. These differences suggest that the TDP tumor classification is a potent predictor of treatment response, irrespective of tumor type. Importantly, SSG and TDP group 1, prevalent in both LUAD and LUSC, share similar clinical, prognostic, transcriptional, mutational, and biological features. It should be noted that the drug susceptibility analysis based on CCLE data included only six LUAD cell lines, which limits the statistical power and raises the possibility of false-positive associations. Therefore, the results should be interpreted as preliminary and hypothesis-generating. Further validation in larger datasets and experimental models is essential to confirm these findings. Thus, classifying TDP tumors into six distinct categories based on molecular determinants holds clinical significance, potentially guiding adjuvant chemotherapy benefits.

In summary, our findings significantly advance the classification of TDP tumor genomic complexity, delineating the molecular features of different TDP groups, elucidating the underpinnings of diverse pathologies and prognoses, and highlighting the clinical implications of distinct TDP groups in adjuvant chemotherapy. Despite these promising findings, several limitations should be acknowledged. First, the analyses were primarily based on retrospective datasets, which may introduce bias. Second, the drug susceptibility analysis was limited by the small number of LUAD cell lines in the CCLE dataset, which may affect the robustness of the results. Third, functional and mechanistic validation of the TDP subtypes and their association with drug response is lacking. Future studies should address these limitations through larger, prospective cohorts and experimental validation.

## Conclusion

We conducted a systematic analysis of the molecular characteristics of TDP, establishing a link between TDP, clinical pathology, therapeutic response, and drug sensitivity. Our findings indicate that patients with a smaller span size exhibit a better therapeutic response and a more active immune system. This insight lays the groundwork for a deeper exploration of the roles TDP plays in cancer, the deep exploration in TDP by using single cell genome data with single cell transcript data might improve the explanation of difference between heterogenous TDP cell types. Moreover, TDP classification would help identify patients who could benefit from chemotherapy.

## Code availability

The code of this manuscript was deposited in Github, the website is https://github.com/jacklee2thu/Comprehensive-multi-omics-analysis-of-tandem-duplicator-phenotypes-in-non-small-cell-lung-cancer.

## Data Availability

The datasets generated and/or analyzed during the current study are available in the UCSC Cancer Genomic Browser repository and CCLE repository. Pertinent masked CNV data, gene expression data (FPKM value), somatic mutation data, clinical data, including relapse events and timelines for TCGA LUAD patients, were sourced from the UCSC Cancer Genomic Browser https://xenabrowser.net/datapages/?cohort=GDC%20TCGA%20Lung%20Adenocarcinoma%20(LUAD)&removeHub=https%3A%2F%2Fxena.treehouse.gi.ucsc.edu%3A443. These data for TCGA LUSC patients were deposited at https://xenabrowser.net/datapages/?cohort=GDC%20TCGA%20Lung%20Squamous%20Cell%20Carcinoma%20(LUSC)&removeHub=https%3A%2F%2Fxena.treehouse.gi.ucsc.edu%3A443. The cell lines data and drug resistance data were source from https://xenabrowser.net/datapages/?cohort=Cancer%20Cell%20Line%20Encyclopedia%20(CCLE)&removeHub=https%3A%2F%2Fxena.treehouse.gi.ucsc.edu%3A443.

## Footnotes

^1^ https://xenabrowser.net/datapages/

^2^ http://genome.ucsc.edu/

^3^ https://portals.broadinstitute.org/ccle/data
