# Supplementary material for: Comprehensive multi-omics analysis of tandem duplicator phenotypes in non-small cell lung cancer
Source: Front Med (Lausanne). 2025 Jun 4;12:1556840. doi: 10.3389/fmed.2025.1556840 (PMC12174438; doi:10.3389/fmed.2025.1556840)
Supplement: Supplementary file 2 [file Image_1.pdf]

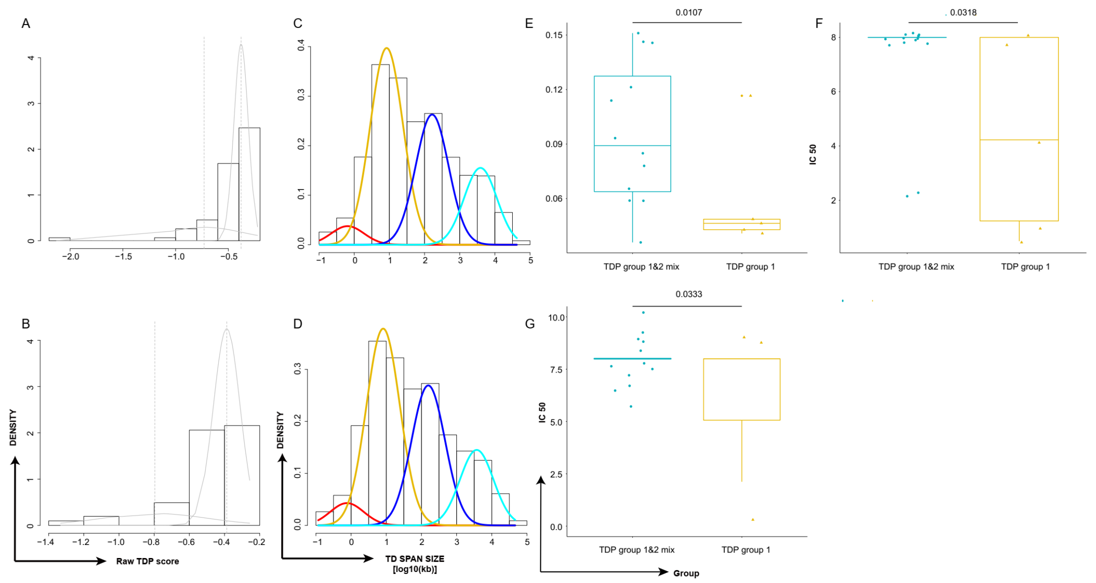

Supplement Figure 1 Drug Sensitivity Across Different TDP Groups.

(A-B) Density plots display the raw TDP scores for CCLE LUAD (A) and LUSC (B).

(C-D) Density plots depict the TD span size (log10 transformed) for CCLE LUAD (C) and LUSC (D).

(E-F) Charts illustrate the distribution of IC50 values for LUAD cell lines treated with Lapatinib (E) and Panobinostat (F) within TDP group 1 and TDP group 1/2 mix.

(G) The graph shows the distribution of IC50 values for LUSC cell lines treated with AZD6244 in TDP group 1 and TDP group 1/2 mix.
